# Investigation of integrated time nanosecond pulse irreversible electroporation against spontaneous equine melanoma

**DOI:** 10.3389/fvets.2024.1232650

**Published:** 2024-01-30

**Authors:** Chris C. Fesmire, Bridgette Peal, Jennifer Ruff, Elizabeth Moyer, Thomas J. McParland, Kobi Derks, Erin O’Neil, Carrie Emke, Brianna Johnson, Shatorupa Ghosh, Ross A. Petrella, Matthew R. DeWitt, Timo Prange, Callie Fogle, Michael B. Sano

**Affiliations:** ^1^Bioelectricity Lab, UNC/NCSU Joint Department of Biomedical Engineering, Raleigh, NC, United States; ^2^Department of Clinical Sciences, NC State College of Veterinary Medicine, Raleigh, NC, United States; ^3^Clinical Studies Core, NC State College of Veterinary Medicine, Raleigh, NC, United States; ^4^Department of Molecular Biomedical Sciences, NC State College of Veterinary Medicine, Raleigh, NC, United States

**Keywords:** focal ablation, pulsed field ablation, INSPIRE, skin cancer, clinical trial

## Abstract

**Introduction:**

Integrated time nanosecond pulse irreversible electroporation (INSPIRE) is a novel tumor ablation modality that employs high voltage, alternating polarity waveforms to induce cell death in a well-defined volume while sparing the underlying tissue. This study aimed to demonstrate the *in vivo* efficacy of INSPIRE against spontaneous melanoma in standing, awake horses.

**Methods:**

A custom applicator and a pulse generation system were utilized in a pilot study to treat horses presenting with spontaneous melanoma. INSPIRE treatments were administered to 32 tumors across 6 horses and an additional 13 tumors were followed to act as untreated controls. Tumors were tracked over a 43–85 day period following a single INSPIRE treatment. Pulse widths of 500ns and 2000ns with voltages between 1000 V and 2000 V were investigated to determine the effect of these variables on treatment outcomes.

**Results:**

Treatments administered at the lowest voltage (1000 V) reduced tumor volumes by 11 to 15%. Higher voltage (2000 V) treatments reduced tumor volumes by 84 to 88% and eliminated 33% and 80% of tumors when 500 ns and 2000 ns pulses were administered, respectively.

**Discussion:**

Promising results were achieved without the use of chemotherapeutics, the use of general anesthesia, or the need for surgical resection in regions which are challenging to keep sterile. This novel therapeutic approach has the potential to expand the role of pulsed electric fields in veterinary patients, especially when general anesthesia is contraindicated, and warrants future studies to demonstrate the efficacy of INSPIRE as a solid tumor treatment.

## 1 Introduction

Equine melanoma occurs in approximately 80% of gray horses (1, 2). The increased incidence of melanoma in these horses and their gray coat color is associated with a gene mutation (STX17) (3–5). Melanocytic tumors may arise in this population via increased proliferation of dermal melanocytes due to duplication of the STX17 gene (3, 4) or more broadly in horses as a consequence of disrupted melanin metabolism resulting in increased formation and activity of resident melanoblasts (6, 7). Some tumors exhibit rapid growth and malignant properties immediately, however, the majority exhibit slow growth over many years with little disease progression (7). These slow growing tumors have the potential to undergo a malignant transformation followed by a sudden rapid growth phase (7, 8). While most melanomas initially present as benign (9, 10), approximately two-thirds are thought to become malignant with the ability to become systemically metastatic (5, 11, 12). It is therefore recommended that all visible and accessible masses be addressed via some form of treatment (10).

Diagnosis is typically based on the physical appearance and location of the neoplasm (6, 7) and supplementary histological analysis can be conducted to assess the degree to which malignant invasive behavior can be expected (13). Equine melanocytic tumors commonly present as solitary discrete or coalescing subcutaneous masses ranging in size from smaller than 1 cm to over 20 cm in diameter (7). They typically arise on the ventral surface of the tail, the perineal skin, and the genitalia, but may also form near the ears and eyelids, on the head and neck, or in internal locations (7). Larger tumors may ulcerate and may eventually lead to physical obstruction of the anal sphincter and genitalia causing dyschezia, dysuria, and dystocia (7).

When possible, melanomas are treated by surgical or laser excision (6, 7, 13, 14) which does not appear to affect continued growth of distal tumors (3, 15). Recurrence may occur if wide margins cannot be achieved and multiple procedures may be necessary to adequately address the disease (7). Cryotherapy (13), chemotherapeutic injections (16, 17), and immunotherapies (18, 19) are among the options when surgery is contraindicated. In cryotherapy for melanoma, the tumor is rapidly frozen via a surface applicator which has been cooled with liquid nitrogen, with applicators internally cooled with liquid or gas (20), or by directly pouring liquid nitrogen on the tumor (21). Multiple rapid freeze - slow thaw cycles are conducted to achieve maximal effect (22). Tissue injury arises from the formation of ice crystals which disrupt the cell membrane (23, 24), disruption of microvasculature (24, 25), and induction of apoptosis due to stress induced during the freezing process (26, 27). While effective for small superficial tumors, it can be challenging to adequately cool large tumors and multiple treatments may be necessary to fully address the disease.

Approaches administering cisplatin and carboplatin achieve a therapeutic effect by cross-linking DNA (3). Injections of these chemotherapeutics are typically administered over a series of four visits at a concentration of 10 mg/mL at two week intervals to administer 1 mg/cm^3^ of the chemotherapeutic to each tumor (16). Similarly, biodegradable beads containing cisplatin (1.6 mg/bead) can implanted into tumors, typically following surgical or laser debulking (17). Both approaches have similar efficacy achieving 100% local control up to two years following treatment (16, 17). There is some risk of acute local reactions to these treatments (16) and because these platinum based agents are carcinogenic and mutagenic, strict safety protocols must be followed during all phases of treatment and follow up (3, 28).

Immunotherapies are an emerging class of therapies which aim to reverse tumor mediated immune suppression or enhance anti-tumor activation of the immune system (3). Interleukin-12 (IL-12) and Interleukin-18 (IL-18), are a class of cytokines associated with the suppression of angiogenesis (29), production of interferon-gamma (20), induction of apoptosis in cancer cells (30), and the activation of cytotoxic T cells and natural killer cells (20, 31). Intra-tumoral injection of DNA vectors or plasmids encoding for these genes resulted in a 20–29% reduction in tumor volume with equine cytokines (32, 33) and a 59% reduction for plasmids encoding for human cytokines (34). Autologous whole-cell vaccines attempt to elicit a similar immune response by surgically implanting tumor samples which have been frozen or irradiated after excision (35) or genetically modified to produce immunogenic proteins (36) and have shown promise in early feasibility studies. Similarly, DNA vaccines encoding for human tyrosinase (e.g., Oncept) which is overexpressed in equine melanoma have been demonstrated as safe to use (37). However, there exists only anecdotal evidence of their efficacy in equine patients (38) and this approach fails to extend progression free survival, disease-free interval, or median survival times in canines (39), limiting enthusiasm for this approach (40).

**Figure 1:**
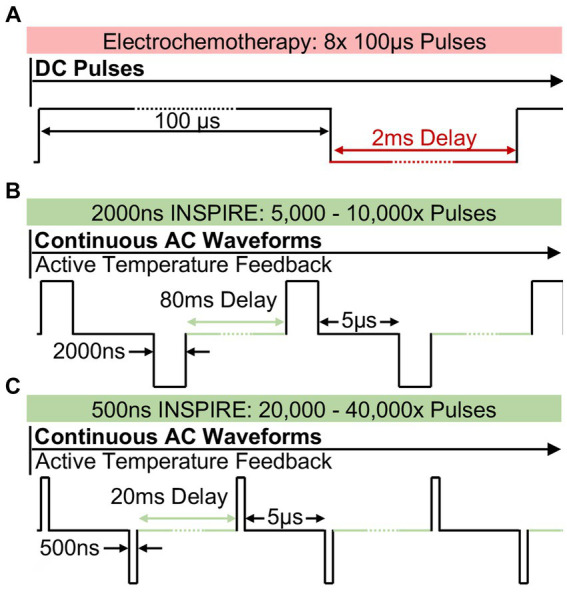
Pulsed electric field strategy utilized in ECT and in INSPIRE **(A)** Typical electrochemotherapy protocols administer 8 × 100 μs mono-polarity pulses which induce intense muscle stimulation. INSPIRE treatments administer an alternating polarity waveform consisting of either **(B)** 2,000 ns or **(C)** 500 ns pulses which are administered continuously. The integrated time, calculated as the pulse duration times the total number of pulses was fixed to either 0.01 s or 0.02 s in this study.

Given the challenges with existing approaches, better treatment options for larger tumors and those within proximity of vital structures would be welcome. This is particularly true for treatments which are synergistic with systemic immunotherapies and those that circumvent the need for general anesthesia. Focal ablation techniques such as electrochemotherapy (ECT) (41, 42) and irreversible electroporation (IRE) (43) have the potential to fill this clinical need if clinical and technical challenges with these approaches can be addressed. ECT combines the focal application of high voltage pulsed electric fields on the order of 100 μs–10 ms (Figure 1A) and chemotherapeutic agents. In this approach, the chemotherapeutic agent is injected into the tumor, and the electrical pulses enhance cellular uptake by temporarily increasing the permeability of the cell membrane to large molecules (44). Transient reduction in vascular perfusion due to the pulsed electric fields helps retain the chemotherapeutic in the treatment zone (45) further enhancing transport of the therapeutic agents into cells. This results in the induction of a number of cell death pathways and release of a number of damage-associated molecular patterns (46, 47) which may enhance recognition of the tumor by the immune system (48). In veterinary oncology, ECT is an emerging approach for the treatment of dermal malignancies including equine skin tumors (1) along with feline and canine sarcomas (49, 50). Unlike ECT, IRE forgoes the chemotherapeutic agent and employs the pulsed electric field for a monotherapeutic lethal effect (51). In this approach, additional pulses (typically 90-100x) 80–100 μs in duration (52) are administered to permanently disrupt cell membranes and induce cell death. In veterinary studies, IRE has been used to reduce tumor volumes in canine patients with intracranial gliomas (53) and soft tissue sarcoma (54). A noted benefit of IRE is that it can be used for larger infiltrative tumors, which are difficult to treat with existing veterinary surface applicators employed during ECT. IRE is also a promising tumor ablation approach in humans with clinical trials in kidney (55), pancreatic (56), and bone tumors (55). The consensus of these trials is prolonged survival times compared to standard of care.

Despite the successful clinical utilization of electroporation-based therapies (ECT and IRE), a common challenge remains; the high voltage pulses induce intense muscle contractions (57, 58). In human clinical trials, patients receive a combination of paralytics (59) and cardiac synchronization (59, 60) to address intraoperative movement due to the intense muscle stimulation. For horses, heavy patient sedation and general anesthesia are typical (49) as the patients react unfavorably to the intense muscle stimulation. General anesthesia is inherently risky in horses (61, 62) as it increases the overall risk of morbidity and mortality from fractures (63–65), cardiovascular failure (63–65), respiratory complications (airway obstruction, poor ventilation, hypoxia, etc.) (64), postanesthetic myopathy (63, 64, 66), and abdominal complications (peritonitis, colitis, etc.) (63, 64).

To address these challenges, recent work has focused on using ultrashort (<2 μs) alternating polarity (positive and negative) electric pulses delivered in a rapid burst (67). These bi-polar pulses have demonstrated the ability to reduce muscle contractions in porcine models (58) and in human clinical trials (21). They also reduce the potential for electrical arcing (68) when compared to ECT or IRE protocols. However, early investigations indicated that these waveforms were less efficient than IRE treatments when matched electrical doses were administered (69, 70) as shorter pulses resulted in smaller treatment volumes (71). Therefore, there has been a clinical tradeoff between maximum treatment volume, which favors longer pulse widths, and reducing muscle contraction, which favors short pulse widths. Integrated-time nanosecond pulse irreversible electroporation (INSPIRE) is designed to overcome treatment volume limitations. In INSPIRE, bi-polar 500 ns to 2,000 ns pulses (Figures 1B,C) are administered until a target integrated time, calculated as the sum of all pulse durations delivered, is achieved (72). This integrated time, or electrical dose, has been shown to be a critical factor affecting the efficacy of these treatments, with shorter pulses requiring a greater integrated time to achieve similar efficacy as longer pulses (72). By tuning treatment parameters, muscle contractions can be reduced (by delivering shorter pulses) while maintaining a clinically relevant treatment volume (by increasing integrated time).

Based on *in vitro* data (71, 73) showing similar efficacy of INSPIRE and IRE treatments at physiological temperatures and *ex vivo* data (74, 75) demonstrating the ability to produce clinically relevant treatment zones, it was hypothesized that INSPIRE may be a safe and effective treatment against cutaneous tumors between 1 and 5 cm in diameter. Equine melanoma was identified as the initial clinical target for this technique based on the clinical need, ease of applying the treatment, and the potential for long-term visual follow up for treatment confirmation.

## 2 Methods

### 2.1 Patient recruitment criteria

Equine patients (Table 1) were recruited into an IACUC-approved (19-053-O) study at the North Carolina State University College of Veterinary Medicine. Inclusion criteria included the presentation of at least three discrete tumor nodules suspected of being melanoma, patient health was adequate to handle sedation, and that patients were not currently receiving any other forms of treatment for melanoma. Horses having received previous treatments (e.g., surgery, cryotherapy) were admitted. Horses underwent systematic physical examination that included examination of heart rate, respiratory rate, rectal temperature, mucous membranes and auscultation of the lungs, heart and abdomen for borborygmus. Informed written consent for this therapy was obtained from owners prior to enrollment. Following an initial visual screening, patients were admitted to the study. Before treatment, candidate tumors were identified, numbered, measured, and photographed. For each tumor, the length (*l*), width (*w*), and depth (*d*) from the tumor apex to the skin were measured with digital calipers. Tumor volumes (*v*) were calculated as an ellipsoid withvolume calculation $v=\frac{4}{3}\pi \;\left(\frac{l}{2}.\frac{w}{2}.d\right)\text{.}$ An additional tumor was resected for histological confirmation of disease. In patients with multifocal disease, only a subset of the tumors were identified, measured, and accounted for across the trial. As this study utilized client-owned animals, tumors that were likely to be problematic to the patient if left untreated were prioritized. Tumors were identified as problematic if any delay in their treatment would risk the horse’s ability to pass manure, negatively impact the prognosis for a successful treatment, or if the tumors were causing the horse noticeable discomfort. Initially, 2 to 8 tumors were identified as candidates and treated, leaving the remainder for treatment during subsequent visits. For each patient, at least one tumor was left untreated to serve as an internal control. A total of 8 patients were admitted into the trial. Two patients were lost to follow up before their first recheck, yielding a total of 6 patients (Table 1), representing 32 treated and 13 untreated control tumors. Histology confirmed melanocytic tumors across the six patients meeting the inclusion criteria. Patients returned twice following the initial treatment at 21–49 day intervals, at which time the tumors were re-measured.

**Table 1:** Summary of horses enrolled in the study.

Horse	Location	Breed	Age	Sex	Treated tumors	Untreated tumors	Follow up time (Days)	
1	Tail	Percheron	19	F	4	3	22	21
2	Tail/Perianal	Quarter Horse	14	F	8	2	36	49
3	Tail	Thoroughbred	18	F	6	2	29	42
4	Tail/Perianal	Andalusian	16	M	5	2	25	21
5	Tail	Andalusian	17	M	4	2	34	33
6	Tail	Lusitano	22	F	5	2	27	21

All tumors were melanocytic tumors and located either on the tail or perianal region. A full accounting of the tumors and the treatments administered is presented in Supplementary Table 1.

Owners obtained photographs of the treatment sites daily for one week and weekly for 12 weeks following treatment. Across three visits, 3 to 8 tumors were treated in each patient. Complete responses were defined as absence of measurable tumor mass at the time of recheck. When complete responses were observed following a single treatment, a secondary cohort of tumors, if present, were treated.

### 2.2 Anesthetic protocol

All horses were treated with INSPIRE standing in standard veterinary stocks. Sedation was administered to effect to achieve a standing position without obvious signs of agitation such as persistent tail swishing, weight shifting, or kicking. Prior to treatment, horses were sedated with detomidine (0.01–0.02 mg/kg IV) and butorphanol (0.01–0.05 mg/kg IV), and re-dosed as needed to facilitate treatment application (detomidine range: 5–15 mg, butorphanol range: 5–10 mg). Treatment sites were prepared with povidone-iodine scrub and alcohol. When possible, tumors were desensitized by local injection of 2% mepivacaine with 15–50 mL SQ total volume, depending on tumor number and location, into the surrounding tissues. Tumors along the ventral tail were frequently difficult to inject with local anesthetic due to the fibrous nature of the masses.

### 2.3 INSPIRE parameters

INSPIRE treatments delivered a waveform consisting of a positive pulse (P), a 5 μs inter-pulse delay (D), and a negative pulse (N), which was repeated continuously until a target integrated time was administered (Figures 1B,C). The integrated energized time was calculated as the sum of all pulse durations delivered. A 0.02 s integrated time, therefore, contains either 5,000× 2,000 ns pulses or 20,000× 500 ns pulses. The delay between successive waveforms was selected to maintain an equivalent energy delivery rate (100 μs/s) independent of the constitutive pulse widths (P and N) so that transient thermal profiles within the tumors were approximately equivalent between voltage-matched treatment groups. This resulted in repetition delays of 0.04 s (25 Hz) and 0.01 s (100 Hz) for treatments with 2,000 ns and 500 ns pulses, respectively. Tumors were assigned at random to 2,000 ns and 500 ns groups, therefore different tumors on the same horse may have been treated with more than one protocol.

The initial protocol prescribed treatments at 2,000 V with a 0.02 s integrated time. However, some horses exhibited sensitivity or discomfort to treatments at this voltage. If additional local anesthetic did not alleviate this, the voltage was reduced to complete the treatment. To evaluate if this reduction in voltage affected treatment outcomes, tumors were separated for analysis into those which were treated with 2,000 V and those which required a lower voltage (1,000 V). Six tumors were treated with 500 ns pulses at 1,000–1,500 V, three tumors were treated with 500 ns pulses at 2,000 V, seven were treated with 2,000 ns pulses at 1,000 V, and ten were treated with 2,000 ns pulses at 2,000 V. Two additional treatments were administered with 2,000 ns pulses with 0.01 s integrated time at 1,500 V to investigate how reductions in dose and voltage affected outcomes. Thirteen tumors were measured and followed, but were left untreated to act as internal controls. A full accounting of the tumors and the treatments they received is presented in Supplementary Table 1.

### 2.4 Treatment approach and follow up

**Figure 2:**
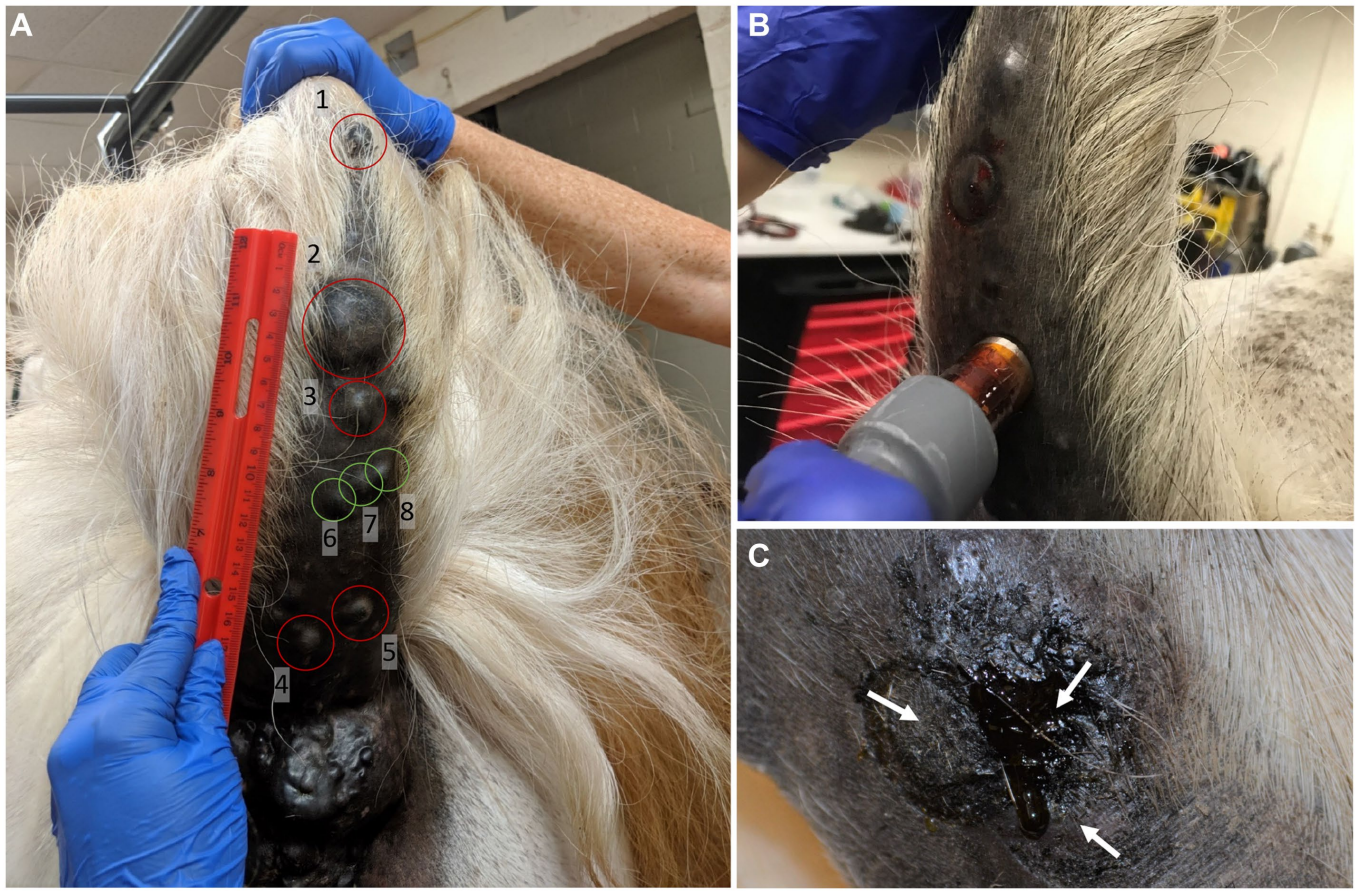
Tumor treatment approach. **(A)** Patient tumors were grouped into treatment (red circles) and non-treatment groups (green circles) prior to the first treatment **(B)** Application of the custom treatment electrode onto tumor **(C)** Tumors that were larger than the electrode’s treatment zone were treated multiple times in an overlapping method. White arrows indicate the center of each treatment application.

**Figure 3:**
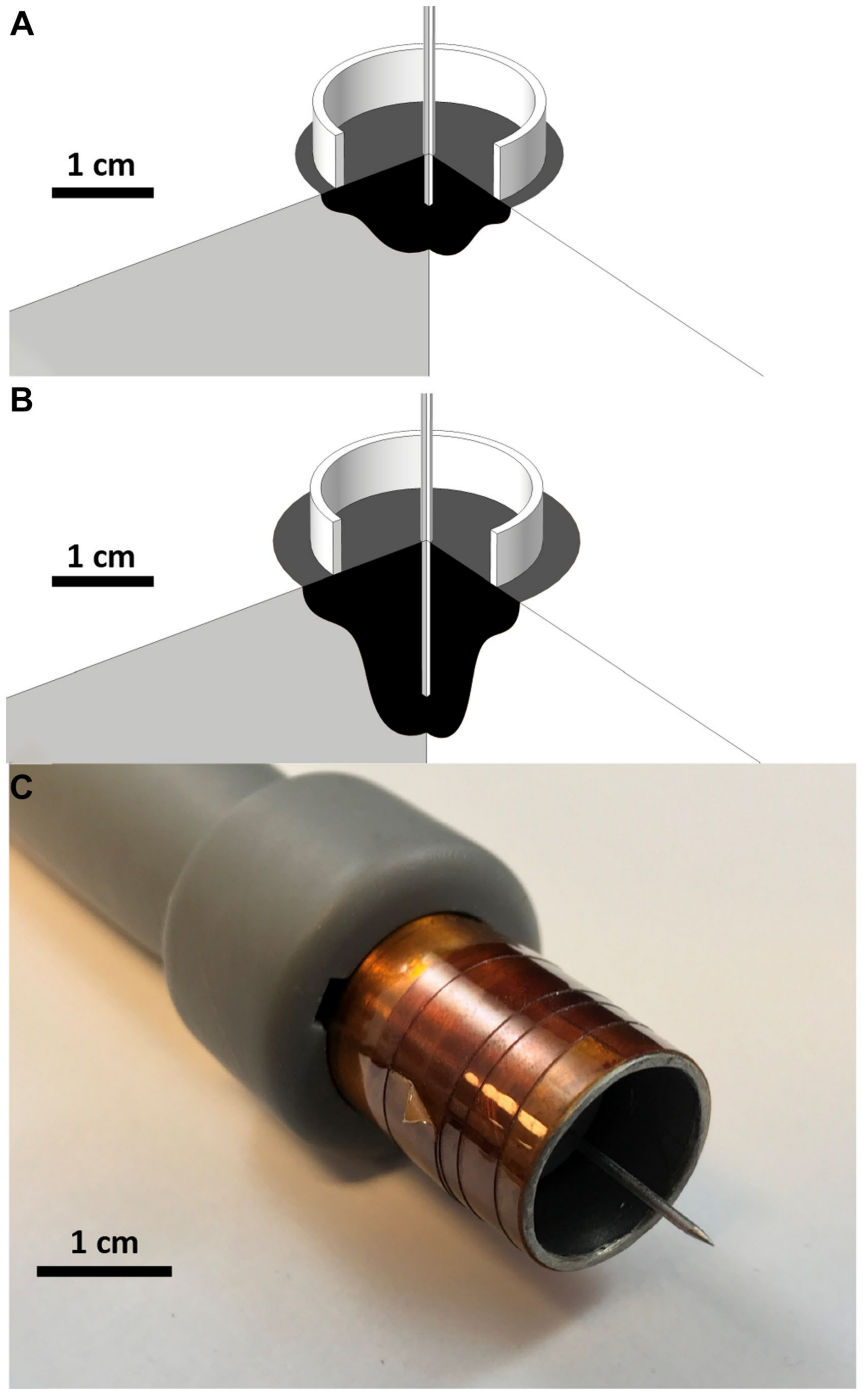
Treatment zone simulation and custom build electrode Treatment zone planning and predictions for various treatment voltages and needle treatment depths using COMSOL Finite Element Analysis software. 270° cross section rotations of **(A)** 2,000 V 5 mm center electrode depth and **(B)** 2,000 V 15 mm Electrode Depths are modeled above. Black regions indicate predicted treatment zones. **(C)** 3D printed and constructed applicator.

Treatment was initiated following measurement of each tumor and the assignment of tumors into control and treatment groups (Figure 2). Simulations (Figures 3A,B) predicted that the treatment zone extended approximately 0.5 cm beyond the outer ring of the applicator. Therefore, for tumors smaller than the applicator diameter (17 mm), the needle was placed directly in the middle of the tumor mass (Figure 2) such that the ring electrode circumscribed the entire volume. Tumors larger than the diameter of the applicator were treated multiple times by overlapping treatment areas, such that the ring made contact with the location of the prior needle puncture, until the complete surface area of the tumor was covered. The needle was inserted until the outer ring electrode made complete contact with the surface of the tumor. In some locations, continuous contact around the circumference was inhibited by the tissue geometry and required application of ultrasound gel to facilitate electrical contact.

Before the full treatment, a ramp up protocol was initiated to ensure proper performance of the generator and applicator and assess patient tolerance of the procedure. In this procedure, a single waveform (one positive and one negative pulse) was administered, and the applied voltage was increased in 250 V steps from 250 V to 2,000 V (Supplementary Figure 1). At this voltage, INSPIRE was administered for 1 s, at which point modifications to the applicator placement, patient sedation, or treatment voltage were made, if necessary. The entire treatment protocol was then delivered up to the target integrated time (0.01 or 0.02 s). Following treatment and recovery, patients were released and prophylactically given 25–30 mg/kg of trimethoprim/sulfamethoxazole (TMS) orally twice a day for a week to prevent local or regional infection at the treatment site. Upon follow-up visits, tumors were measured and photographed. A complete response was defined as the absence of measurable or palpable tumor mass upon the follow-up visit.

A Welch’s t-test was utilized to determine the statistical significance (*p* < 0.05) in tumor size reduction between treatment parameters. A McNemar’s Chi-squared test for paired counts was used to assess if there was a statistical significance (*p* < 0.05) between complete remissions in these groups.

### 2.5 Clinical applicator

A custom electrode applicator was developed to treat the relatively spherical cutaneous melanoma tumors enrolled in this study (Figure 3). A coaxial, or ring-and-needle design, was adapted from a similar geometry used in *in vitro* studies (71, 73, 76). Finite element numerical analysis (COMSOL Multiphysics, COMSOL Inc., Los Altos, CA) was conducted to optimize geometry, inform treatment planning, and validate the *in vivo* results. Briefly, a 2D axisymmetric simulation consisting of a perfect electrical conductor in the coaxial geometry and homogenous, cutaneous skin tissue [conductivity: 0.491 S/m at 100 kHz (77)] was conducted. Simulated treatment zones were defined as the volume of tissue exposed to electric fields of 500 V/cm or greater (71, 78, 79) (Figure 3). The geometry was optimized parametrically with the goal of achieving a 2 cm diameter treatment zone while minimizing the overall treatment voltage.

The final design selected for fabrication and use in this study consisted of a 17 mm diameter stainless-steel ring designed to rest flush on the skin surface and a 1 mm diameter central needle to penetrate the tumor. To account for patient-to-patient variability in tumor dimensions, the penetration depth could be set between 0.1 cm and 3.0 cm. Simulations (Figures 3A,B) indicated that the treatment zone extended approximately 0.5 cm from the distal tip of the needle; Therefore, the needle penetration depth was adjusted between treatments to ensure the applicator reached the distal margin of the tumor to achieve an approximate 0.5 cm margin. The electrode applicators were assembled into a 3D printed (Form2, Form Labs Inc. Summerville, MA) handle which incorporated the necessary elements to keep the needle centered and to provide physical isolation between the ring electrode and the needle electrode (Figure 3C).

## 3 Results

### 3.1 Procedural observations and findings

Single treatments with the coaxial electrode produced a treatment zone which was circular at the tissue surface and extended into the tissue in the direction of the central needle. The width of the treatment zone narrowed as it approached the distal aspect of the needle as depicted in numerical simulations (Figures 3A,B). Tissue necrosis (Supplementary Figure 2b, 1 day post) was centered where the needle was inserted and became less severe with increasing distance from the tissue surface. This necrotic zone extended a few millimeters beyond the diameter of ring electrode for 2,000 V treatments. Healthy granulation tissue and new epithelium typically formed around the necrotic core (Supplementary Figure 2c, 22 days post) the necrotic core and the wound typically continued to granulate and contract (Supplementary Figure 2d, 43 days post).

Local muscle stimulation was observed during the onset of some treatments, the extent of which was pulse-width, voltage, patient, and location dependent, but generally subsided as the treatment progressed (Supplementary video 1). Administration of additional local anesthetic was observed to mitigate this muscle stimulation but was not directly quantified. Some standing patients continued to present sensitivity or discomfort in response to the electrical stimulation after additional administration of local anesthetic. This presented as tail swishing, local muscle twitching, weight shifting, head movement, or other changes in alertness. In these instances, the voltage was reduced to complete the treatment. Total procedural times (including site preparation, treatment, and recovery) were between 15 and 120 min, depending on the number and size of tumors treated. Energy was delivered for approximately 100 s for each 0.01 s dose treatment (small tumors) and 200 s for each 0.02 s dose treatment (medium tumors). For large tumors requiring multiple overlapping treatments, this time scaled linearly with the number of applications (e.g., 6–12 min for 2x – 4× 0.02 s dose treatments). For the 2,000 V treatments the initial electrical current delivered displayed some tumor-to-tumor variability with initial values ranging between 5A and 25A. This was likely due to variability in the electrode penetration depth and tissue electrical properties. The output voltage from the pulse generator remained stable throughout treatments, independent of this factor (Supplementary Figure 3a). Throughout each treatment, the electrical current increased asymptotically (Supplementary Figure 3b) as the resistance of the tumors decreased (Supplementary Figure 3c) in response to the treatment.

There was occasional static discharge observed when horses leaned against the metal stocks during some treatments. This was alleviated by placing standard clinical towels between the exposed metal and the horse.

### 3.2 Qualitative and observed tumor response

Immediately following treatment, palpation of some tumors was noted to have reduced firmness. 1–2 days post-treatment, tissue necrosis, and mass size reduction were observed via gross appearance in client provided photographs (Supplementary Figures 2, 4, 5). Minor edema and swelling were observed in a subsection of tumors over the first 24 h. In some cases, a viscous black discharge was observed during this period (Supplementary Figure 5b). However, this was not consistent across all cases. Treated tissue typically formed an eschar (Supplementary Figure 2b) overlaying a region of healthy regenerative tissue. This eschar could be removed via vigorous scrubbing (Supplementary Figure 2c), however, they typically resolved into granulation tissue after 9–17 weeks with no further tissue necrosis observed (Supplementary Figures 2d, 4c, 5d).

### 3.3 Quantitative tumor response

**Figure 4:**
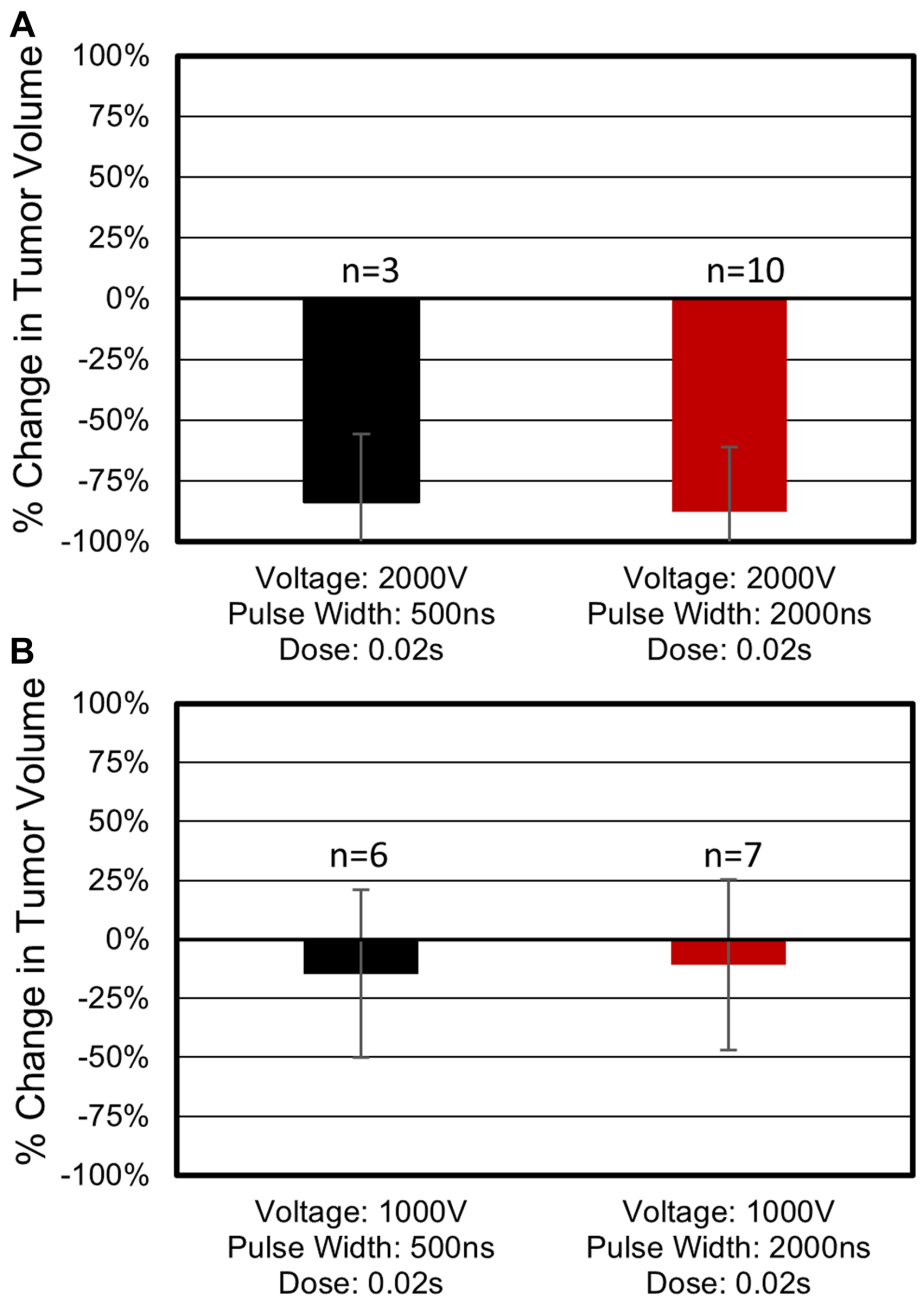
Tumor response as a function of pulse width and voltage. **(A)** Treatments administered at 2,000 V. Three tumors treated with 500 ns, 2,000 V, 0.02 s protocols reduced in volume on average by 84% with 33% (1 of 3) of tumors showing full regression upon the patient’s final follow up visit. Ten tumors treated with 2,000 ns, 2,000 V, 0.02 s protocols showed an 88% reduction with 80% (8 of 10) of tumors indicating a complete response. **(B)** Treatments administered at 1,000 V. Six tumors treated with 500 ns, 1,000 V, 0.02 s protocols which showed 15% reduction in tumor volume. Seven tumors treated with 2,000 ns, 1,000 V, 0.02 s protocols showed an 11% volume reduction. Neither of the 1,000 V treatment groups in **(B)** resulted in a complete response. Average volume changes and standard deviations are shown above.

Treatments with the longest pulses (2,000 ns) and highest voltage (2,000 V) achieved both the largest tumor volume reduction and highest rate of complete responses (Figure 4A). For this specific protocol, tumor volumes reduced on average by 88%, and a complete response rate of 80% (8 of 10) was achieved (Supplementary Figure 6a). In this group, the median tumor volume reduction was 3.50 cm^3^ (range: 0.61–26.09 cm^3^). Only one complete response was observed for equivalent treatments with 500 ns constitutive pulses (2,000 V, 0.02 s) (Supplementary Figure 6b). Tumors in this 500 ns group reduced in volume by an average of 84%, with a median reduction of 2.57cm^3^ (range: 1.56–4.04 cm^3^). Statistical analysis of tumor volume changes between the 2,000 V 500 ns (*N* = 3) and 2,000 V 2,000 ns (*N* = 10) treatments (Figure 4) indicated there was not a significant difference (*p* = 0.83) between these two parameter groups and there was not a statistical difference (*p* = 0.11) in the rate of complete remissions for these parameters.

When 2,000 ns protocols were administered at 1,000 V tumors reduced in volume by 11% (Supplementary Figure 7a) (median: 0.07 cm^3^, range: −0.47 – 1.65 cm^3^). This volume change was significantly smaller than when matched treatments were administered at 2,000 V (*p* = 0.0001). No complete responses were observed for 2,000 ns treatments administered at 1,000 V. For the 500 ns protocols, a treatment voltage of 1,000 V resulted in a reduction in tumor volume of 15% (median 0.04 cm^3^, range: −0.15 – 0.28 cm^3^). This volume change was significantly smaller than when matched treatments were administered at 2,000 V (*p* = 0.02). No complete responses were observed for 500 ns treatments administered at 1,000 V. These results indicate a significant improvement in treatment efficacy when 2,000 V treatments are administered independent of the pulse width used. A full accounting of each tumor response can be found in Supplementary Table 1.

### 3.4 Untreated tumor response

Thirteen tumors were measured but were left untreated to act as internal controls. Eleven tumors reduced in size with an average volume reduction of 50% for these respective tumors upon the last follow up visit (Supplementary Figure 8). Confounding these results, two control tumors (P3-T4 and P6-T5) increased in size from baseline by 153 and 207% upon the first follow up visit, respectively. Upon the second follow up, P3-T4 had reduced in size, but was still 69% larger than baseline. Tumor P6-T5 continued to grow and increased to 667% of baseline. Excluding these tumors, the remaining control tumors reduced in volume between 0.06 and 3.07 cm^3^ (median: 0.25 cm^3^). A reduction in control tumor volume was not an anticipated outcome. Treatments were not standardized by patient, with patients receiving a combination of different treatment protocols between tumors, making it challenging to attribute this observed response with a particular treatment parameter set or to other clinical factors (e.g., tumor biopsy). Patient 1 and 6 were treated exclusively with 2,000 ns protocols. Three control tumors were tracked for Patient 1 with one reducing in size upon the first follow up visit (22 days post treatment) and the remaining two reducing in size upon the second follow up visit (43 days post initial treatment) (range: 0.06–0.54 cm^3^). One of two control tumors tracked for Patient 6 reduced in size following the first and second follow up. Patients 2–4 were treated with a combination of 500 ns and 2,000 ns protocols (Supplementary Figure 8) and had at least one control tumor which reduced in volume.

## 4 Discussion

In this study, a complete response rate of 80% was found for the INSPIRE protocol with the highest dose and longest pulse durations. This single treatment protocol resulted in an 84% reduction in average tumor volume following a single treatment. While follow-up times in this study were relatively short (60 ± 17 days), these results indicate that INSPIRE may be a useful substitute for surgery in certain locations where proximity of vital structures may make surgery challenging or contraindicated. Surgical resection results in local control in approximately 93% of cases (80), however, local and distant recurrence remains a challenge (6, 15) and multiple surgeries may be necessary to adequately address melanomas (7). Local control following INSPIRE can likely be improved by administering treatments over multiple visits. Since completion of this study, it has become our standard practice to schedule three appointments at one-month intervals to repeat treatments if residual tumor tissue is present or if there is concern about microscopic disease.

The ultrashort alternating polarity pulses administered in this study enabled treatments with mild sedation and local anesthetic while horses were standing in standard surgical stocks. In most treatments, there was no observable muscle stimulation, however, this was dependent on the patient and the location of the tumor. The administration of additional local anesthetic was sufficient to alleviate local muscle activation in most animals. An epidural approach for administration of analgesia and anesthetic may additionally alleviate this response, provided that the tumors are in an appropriate anatomical location. The risks of epidural administration, such as lack of response or recumbency (81), must be weighed relative to the benefits of more widespread analgesia and desensitization as indicated for perianal and tailhead tumor treatment. In this study, we did not administer an epidural because intravenous sedation and injections of local anesthesia were more efficient and practical for an outpatient procedure and found to be sufficient to facilitate application of this therapy to the focal areas selected during the clinical trial. For other regions of the body, intravenous sedation and local or regional anesthesia are the only methods for facilitating application of INSPIRE in standing horses. Mitigating intense muscle stimulation has historically been a challenge in administering traditional electrochemotherapy in equine patients (41, 82). In our experience, traditional electrochemotherapy treatments are unlikely to be tolerated by standing horses due to the induction of intense muscle contractions and administration required complete sedation and general anesthesia to avoid issues with compliance. This muscle stimulation can be highly variable, ranging between local twitching and intense movement of adjacent limbs. Muscle stimulation associated with traditional ECT is attributed to the relatively long duration (100 μs or greater) monopolar electrical pulses administered where the electric field strength required to stimulate muscle tissue is approximately 1 V/cm (83). The need for high voltages (1,000–3,000 V) to achieve therapeutic effect results in a significant volume of normal tissue being elevated above the threshold associated with muscle stimulation. In contrast the electric field strength required to stimulate muscle stimulation with the 2,000 and 500 ns pulses used in this study is approximately 50x to 100x greater (50 to 100 V/cm (83)) resulting in a significantly smaller volume of tissue exposed to field strengths high enough to induce muscle stimulation.

While not quantitatively measured in this study, 500 ns treatments were characterized by less muscle stimulation than 2,000 ns treatments. This study highlights two potential approaches for reducing muscle stimulation: reducing treatment voltage and reducing pulse width. A significant difference was not found between voltage-matched treatments with 500 ns pulses and 2,000 ns pulses. However, a significant difference was found between treatments administered at 1,000 V and those administered at 2,000 V. If patient discomfort is observed, then an appropriate clinical strategy may be to first reduce the pulse width from 2,000 ns to 500 ns before attempting to reduce the treatment voltage. A larger study is warranted to study the effect of pulse width, voltage, and dose in more granular detail. As heating and muscle stimulation are of interest, these could be quantified via the use of thermal measurements (84) or temperature control (73) and the use of accelerometers to measure movement (43, 67) to determine if there is an optimal strategy which minimizes discomfort while achieving maximal therapeutic effect. As treatments using 2,000 V, 2,000 ns pulses and a dose of 0.02 s dramatically reduced overall tumor volumes with a single treatment, these parameters should be considered as a baseline for treating tumors moving forward.

Applicator selection is a critical factor in the success of electroporation-based treatments. The region of tissue affected using surface applicators and needle pairs can be difficult to conceptualize due to the exponential decay in electric field strength around these applicators (85). The intention of the coaxial applicator used in this study (Figure 3) was to simplify the geometry of the treatment zone. The ring component of the applicator left a visible impression on the skin following treatment which assisted in overlapping treatment areas on larger tumors (>2 cm) when multiple treatments were necessary to achieve total tumor coverage. Cells in these overlapping areas received a larger cumulative dose which has been shown to increase treatment efficacy (71, 86) and no deleterious effects were observed in regions where treatments overlapped. Tumors larger than 5 cm can be treated with this overlapping approach; In this scenario, it is recommended to address the tumor progressively over a series of 3 to 4 visits at one-month intervals. Three to five overlapping treatments should be administered each session beginning at one side of the tumor and progressively covering the entire tumor over the series of visits.

While this applicator appears to be highly effective at treating cutaneous tumors, it should be noted that it would be challenging to use for internal or deep-seated tumors. For internal tumors, two or more needle applicators can be utilized to administer INSPIRE treatments, however, additional studies are necessary to determine appropriate treatment parameters for this approach. Similarly, a single needle and grounding pad approach (74, 87, 88), akin to the approach used in radiofrequency ablation, can be used. It should be noted that this approach may stimulate muscle tissue between the applicator and grounding pad and may only be feasible under general anesthesia.

While robust systemic responses remain elusive, locally administered chemotherapeutic (16) and immunotherapeutic (34) approaches achieve partial responses in approximately 90 and 40% of cases, respectively (80). Combinatorial approaches which include INSPIRE treatment may improve overall response rates. This is of particular interest in combination with immunotherapies where antigen release by INSPIRE may enhance tumor recognition leading to a systemic immune response. An abscopal effect, where untreated tumors also shrink in size following treatment of a primary tumor, has previously been reported in both ECT (89) and IRE (90–92) studies and pulsed electric fields have been shown to stimulate adaptive immune response *in vitro* (93) and in small animal (94, 95) models of disease. This study observed a reduction in tumor volume in untreated tumors in 11 of 13 control tumors tracked. A more thorough experimental design and analysis are necessary before drawing conclusions regarding the mechanisms behind this observation or potential clinical implications as pulse parameters were not isolated within or across patients. However, we hypothesize that INSPIRE treatment results in the release of immunogenic tumor antigens leading to an adaptive response. INSPIRE treatments administered a higher electrical dose (0.02 s) than traditional IRE treatments (0.08–0.01 s) which may have resulted in an increase in the quantity of tumor antigens released. Additionally, the INSPIRE treatments were administered at a relatively slow rate (50 μs/s) compared to traditional cardiac synchronized IRE treatments (80–100 μs/s) which may have resulted in smaller temperature changes than would normally be expected for IRE treatments and may have prevented thermal denaturation of any released antigens. Combined, these effects may be responsible for the observed abscopal response.

As an abscopal response was an unanticipated outcome, the requisite immune assays to elucidate the mechanism of this abscopal control of distant lesions were not included in the protocol. Patients with larger tumors received multiple treatments to cover those tumors; some patients had tumors treated with 500 ns and 2,000 ns pulses. Within the patient population, there was no observable evidence of a systemic response or global reversal of the disease state. The question of whether the INSPIRE anti-tumor immune response is regionally constrained is unanswered. It should also be noted that two of the eleven control tumors increased in size. While outside the scope of this study, the results indicate that improving and tuning an adaptive immune response following INSPIRE is a promising avenue for treating multifocal disease in patients. In future studies, it will be important to investigate these immunological effects in animals receiving only a single treatment. INSPIRE administered with active temperature control (71, 73–75, 94) could be used to evaluate if thermal effects have an impact on immunological responses. Similarly, untreated local and distant control tumors could be biopsied to investigate the presence and quantity of activated T-cells at intervals before and after treatments. Similarly, it may be interesting to investigate if INSPIRE can improve the efficacy of checkpoint inhibitors against equine melanoma (96–98).

## 5 Study limitations

This study has several important limitations. This study utilized client-owned animals, some of whom had undergone other prior treatments for melanoma. Scheduling and coordination between the clinic and owners resulted in patient-to-patient variation in the time between successive follow up appointments, and some qualitative assessments utilized client obtained photographs. Disease was confirmed via biopsy of a nearby untreated tumor. While unlikely, it is possible that the tumors included in this study were of a different pathology (e.g., nodular sarcoid, eosinophilic granulomas), future studies should consider conducting needle aspirates of all treated tumors to confirm disease pathologies prior to treatment. A small number of animals were evaluated in this study and two of the eight enrolled were lost to follow up following their first visit. Tumor volumes were tracked across multiple visits, but long term (years) responses have yet to be quantified. Finally, only a small set of possible treatment parameters were investigated (pulse width, dosage, and voltage).

Further investigation of INSPIRE parameters is necessary to optimize treatment outcomes. Treatments did not result in complete control of all tumors, as seen in previous studies utilizing IRE. However, the promising tumor control results seen in a subset of INSPIRE treatments and the positive findings of a potential abscopal effect in untread lesions substantiate further clinical use and studies of INSPIRE for the treatment of surface lesions in equine patients.

## 6 Conclusion

This study demonstrated INSPIRE’s ability to significantly reduce the volume of equine melanocytic tumors. Treatments administered via a coaxial ring and pin electrode were well tolerated by equine patients using a combination of standing sedation and local anesthetic. Treatment parameters consisting of 2,000 V, 2,000 ns waveforms with a treatment integrated time of 0.02 s resulted in an 88% reduction in tumor volume and 80% complete response rate. These results were achieved without the use of chemotherapeutics, the use of general anesthesia, or the need for surgical resection in regions which are challenging to keep sterile. This novel therapeutic approach has the potential to expand the role of pulsed electric fields in veterinary patients, especially when general anesthesia is contraindicated, and warrants future studies to demonstrate the efficacy of INSPIRE for tumor treatment.

## Data availability statement

The raw data supporting the conclusions of this article will be made available by the authors, without undue reservation.

## Ethics statement

The animal studies were approved by North Carolina State University. The studies were conducted in accordance with the local legislation and institutional requirements. Written informed consent was obtained from the owners for the participation of their animals in this study.

## Author contributions

CFo, TP, and MS conceived the experimental design. CFe, RP, MD, and MS oversaw use of INSPIRE hardware, analyzed data, and drafted the manuscript. BP, JR, EM, TM, KD, TP, and CFo conducted INSPIRE treatments. CE and BJ managed study setup, patient recruitment, and coordination. SG assisted in the drafting and editing of the manuscript. All authors contributed to the article and approved the submitted version.
