# Supplementary material for: Investigation of integrated time nanosecond pulse irreversible electroporation against spontaneous equine melanoma
Source: Front Vet Sci. 2024 Jan 30;11:1232650. doi: 10.3389/fvets.2024.1232650 (PMC10861690; doi:10.3389/fvets.2024.1232650)
Supplement: Supplementary file 1 [file Data_Sheet_1.PDF]

Supplementary Material

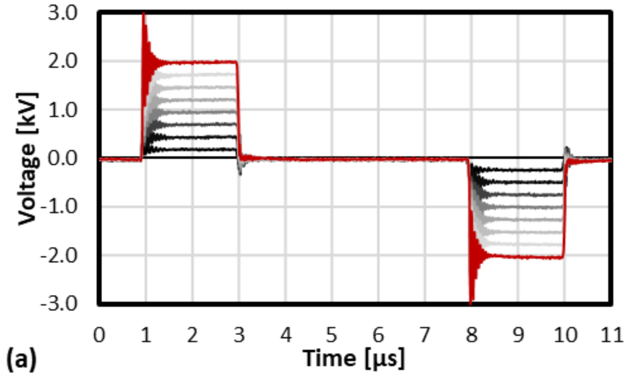

Supplemental Figure 1: Pulse waveforms during the ramp-up procedure. (a) Voltage and (b) current measurements recorded during a typical ramp-up procedure. This ramp up was conducted to confirm adequate contact between the tissue and applicator as well as to cautiously observe the patient's tolerance

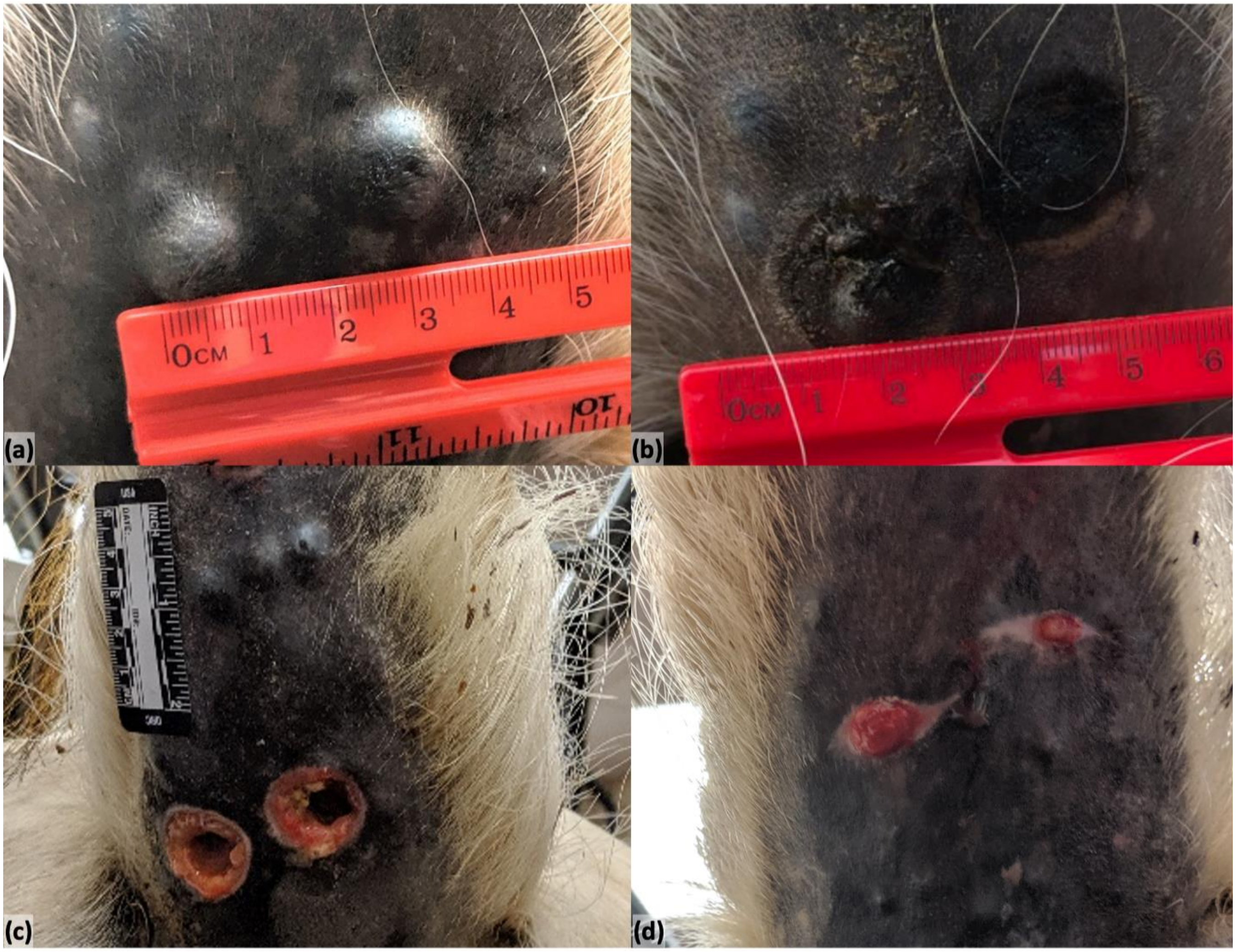

Supplemental Figure 2: Ablation zone and wound progression. Two tumors treated with 2000ns pulses at 2000V and an integrated treatment time of 0.02 seconds, resulting in a total of 5,000 sets of bi-polar pulses delivered to the tumors. (a) Pre-treatment and (b) 1 days post treatment, (c) 22 days post treatment (d) 43 days post treatment.

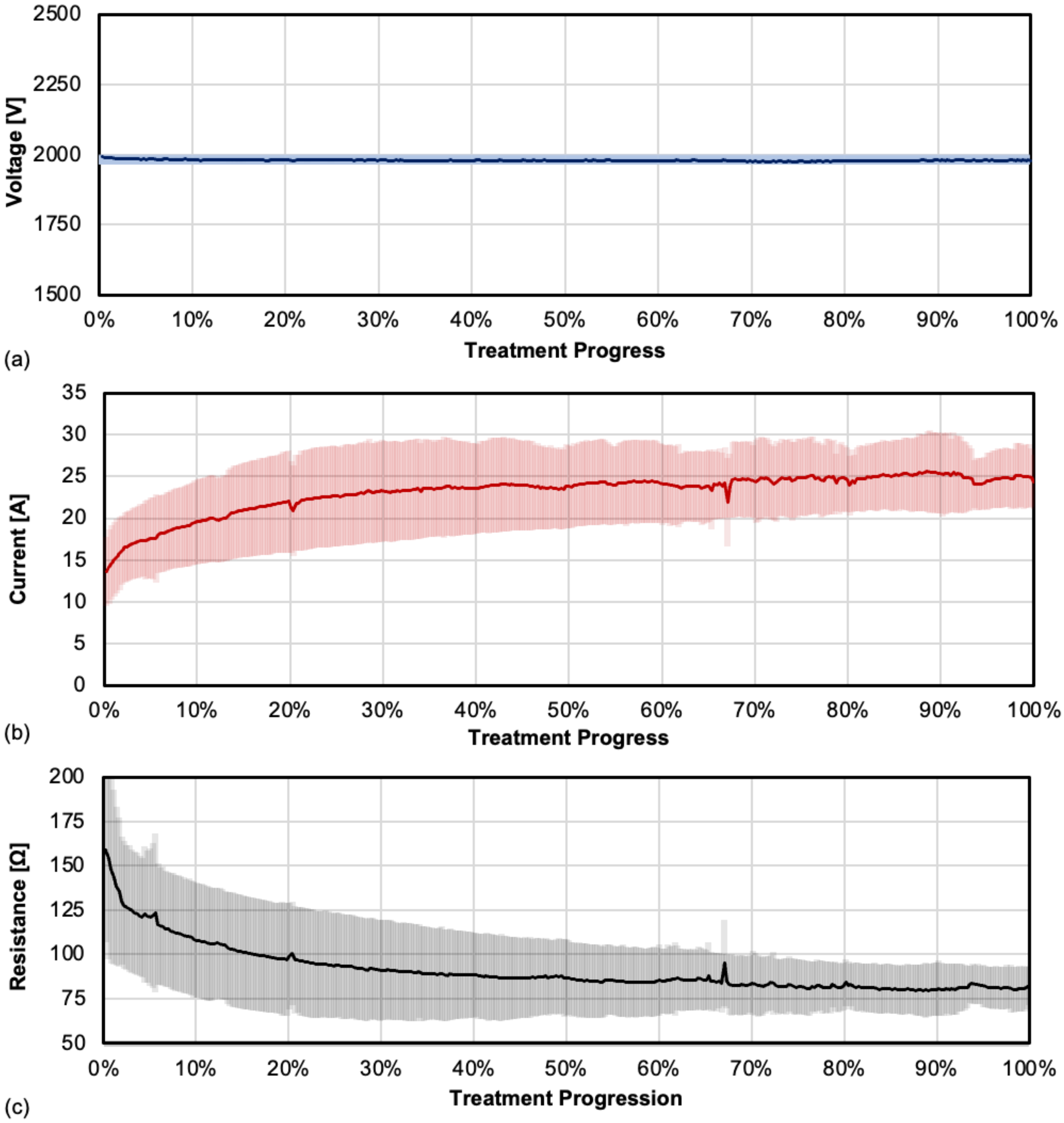

Supplemental Figure 3: Tumors decrease in impedance as treatment progresses resulting in an increase in current. Average (a) voltage, (b) current, and (c) resistance measurements (n=5) recorded during the treatment of a single large tumor (Supplemental Figure 4) presented by Patient 2. Shaded regions represent 1 standard deviation from the mean.

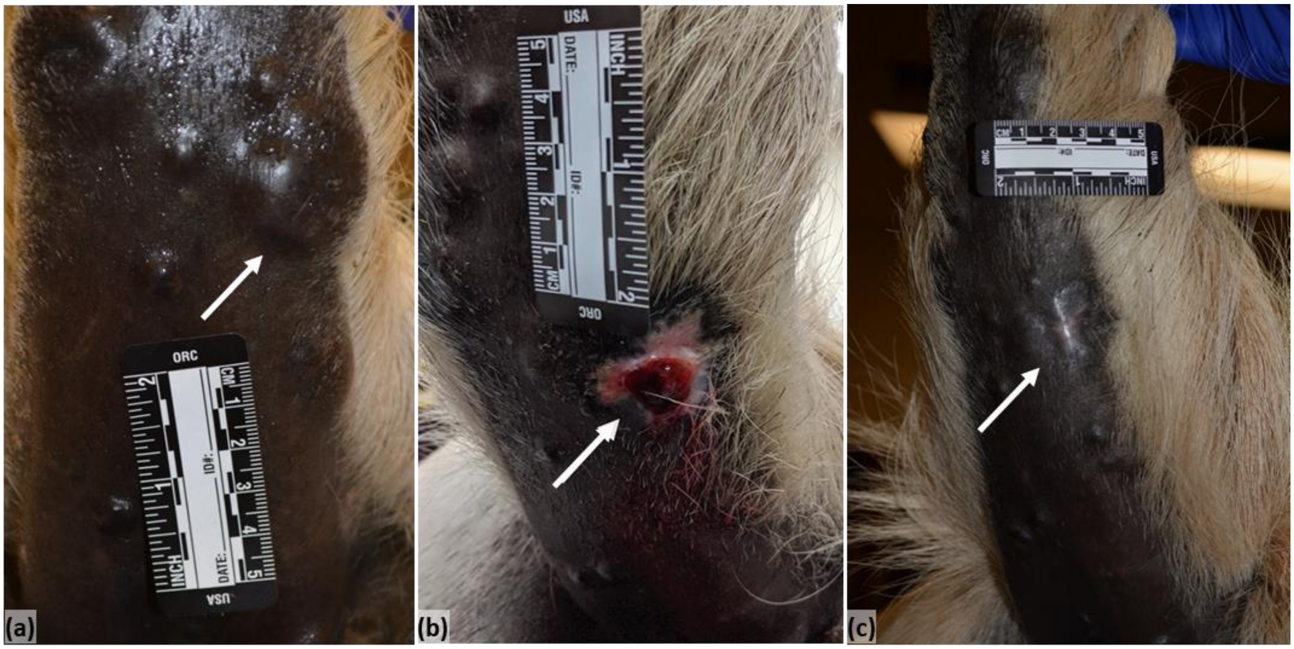

Supplemental Figure 4: Tumor response following a 2000V, 2000ns treatment (a) Initially sized 3.5 x 3.546 x 1.1 cm presented by Patient 2, Tumor #2. To achieve complete tumor coverage, 5 overlapping treatments were administered with a 2000V, 2000ns 0.02s protocol in each location. **(b)** A complete reduction in tumor volume with an exposed cutaneous wound was observed 36 days after treatment. This **(c)** wound resolved by the third visit 86 days later.

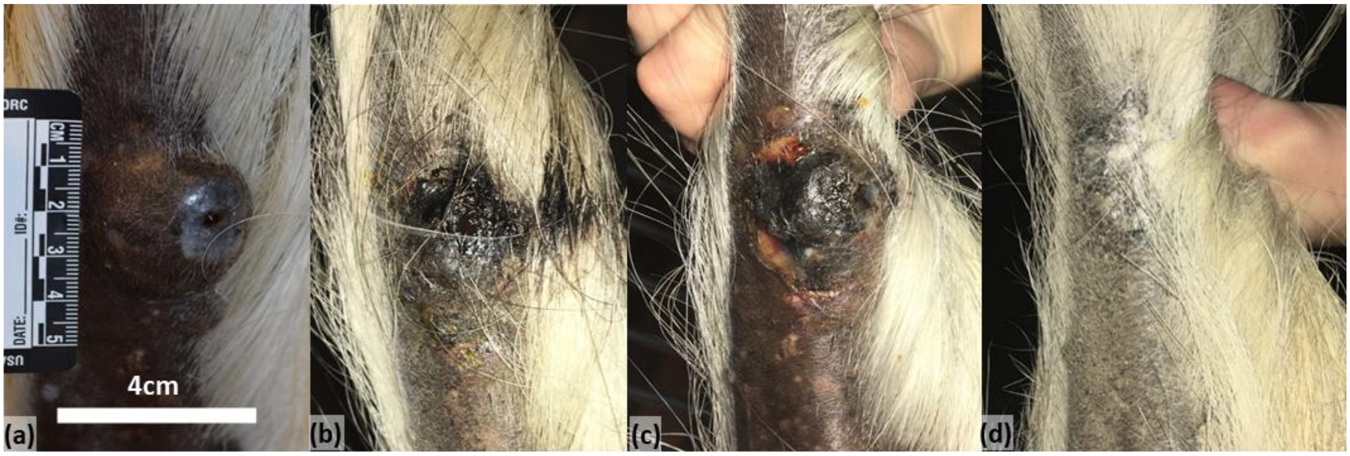

Supplemental Figure 5: Tumor response following a 2000V, 2000ns treatment treatment. A patient presenting a 3.7 x 3.0 x 2.5 cm melanoma tumor treated in 5 locations (middle, right margin, left margin, top margin, bottom margin). (a) Pre-treatment image. (b) 1 day and (c) 2 days after the treatment the tumor showed significant activity and drainage. (d) 122 days after the single treatment no tumor bulk was present and the tumor site had scarred over.

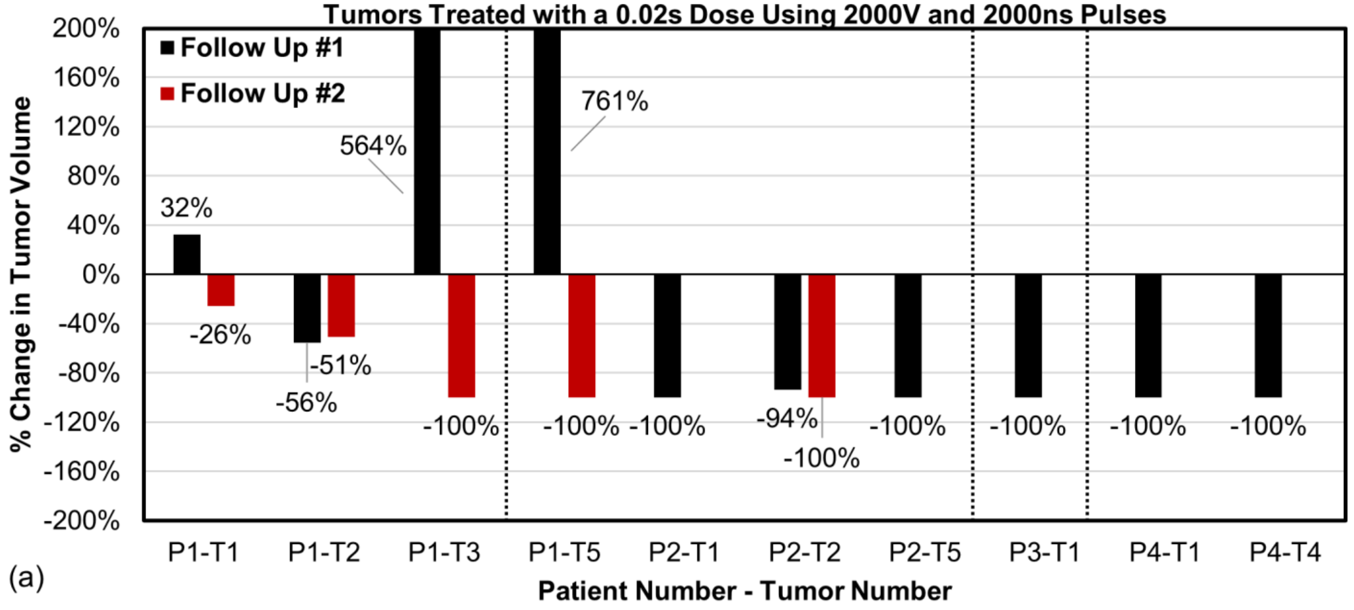

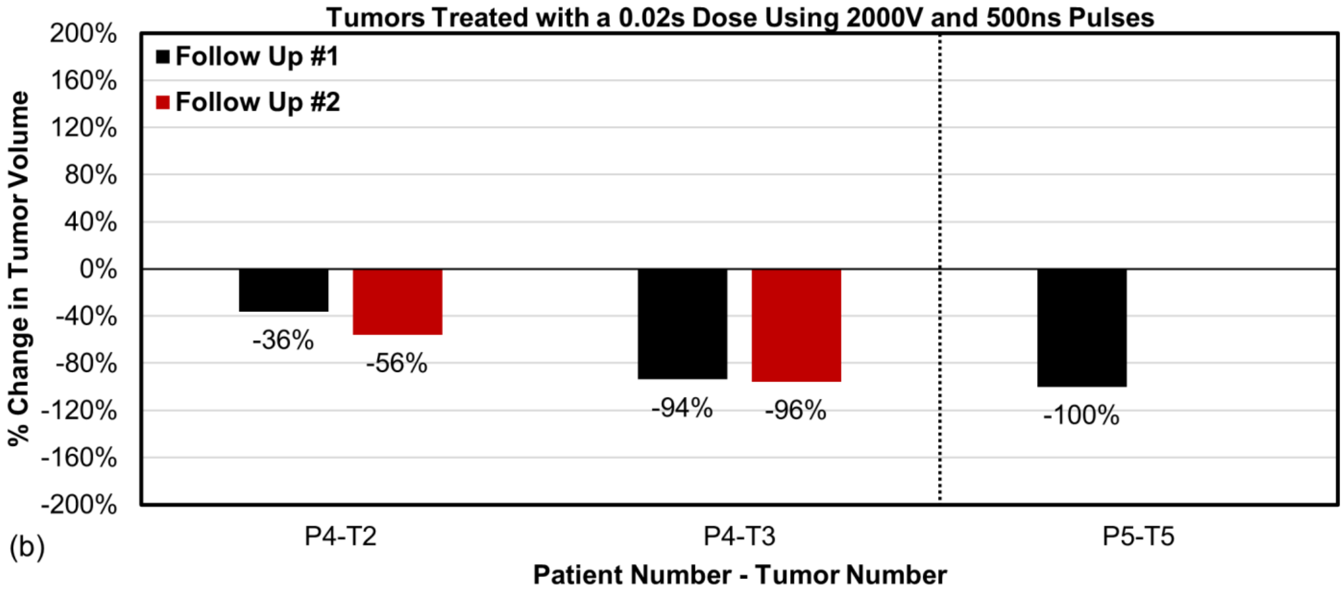

Supplemental Figure 6: Tumor response following 2000V Treatments. Individual tumor response from 2000V treatments consisting of 0.02s dose with (a) 2000ns pulses and (b) 500ns pulses. “P#” represents the patient number and “T#” represents the tumor number as recorded on the patient’s records. Vertical dotted lines separate individual patients.

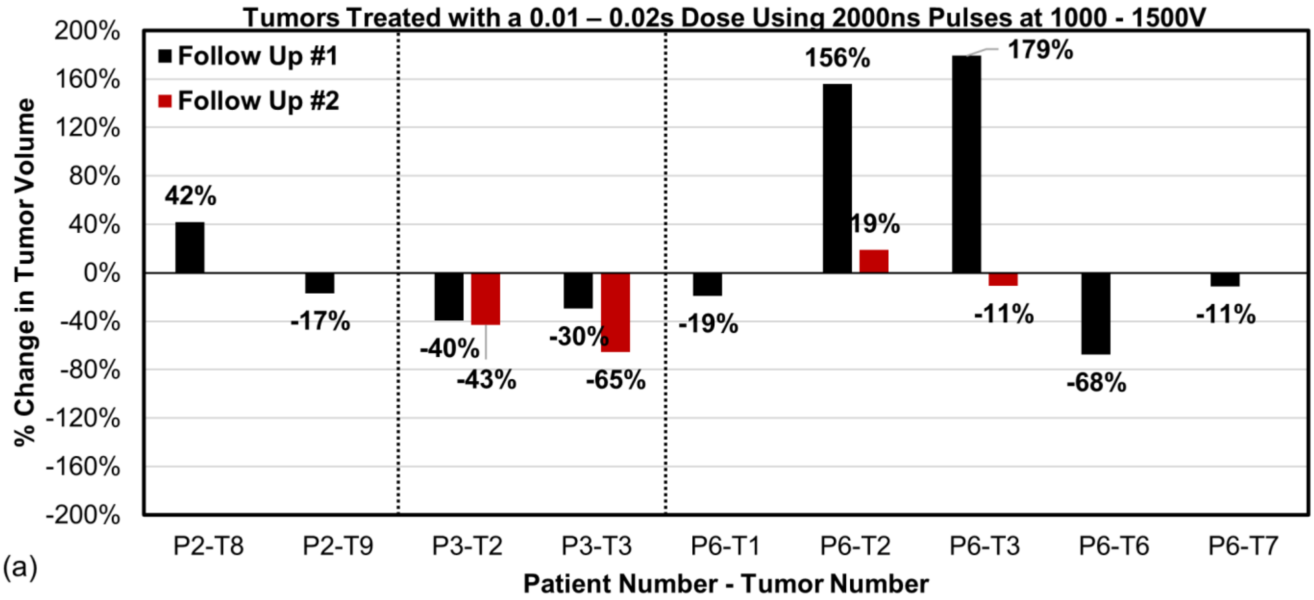

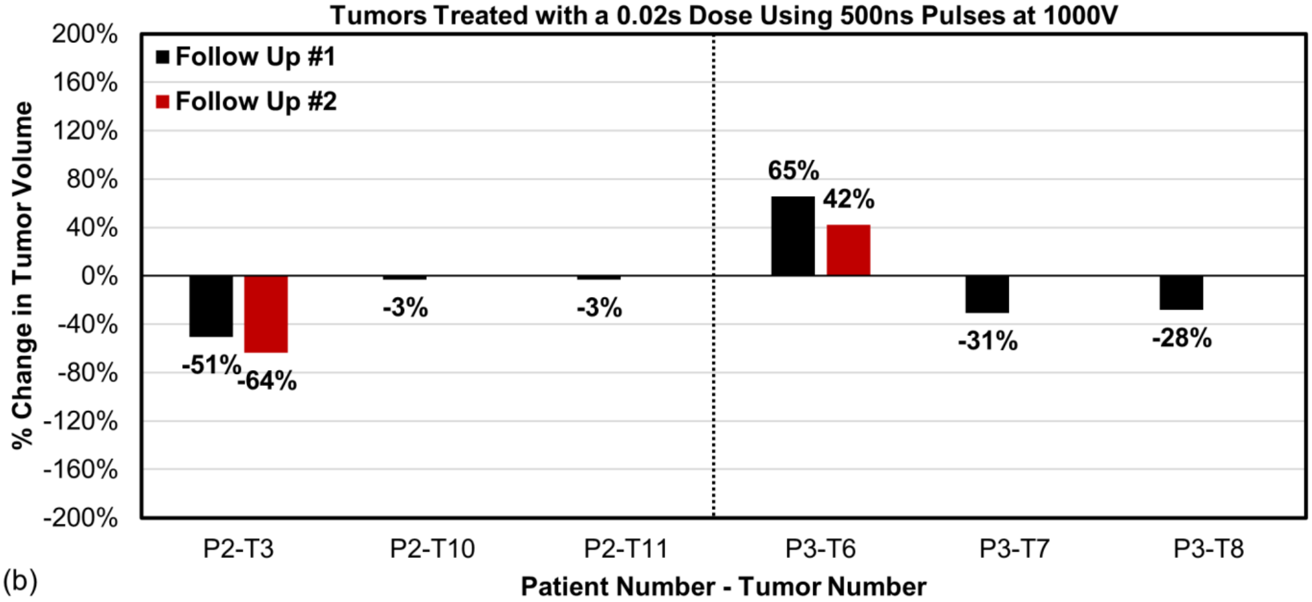

Supplemental Figure 7: Tumor response following lower voltage treatments. Individual tumor response from 1000-1500V (a) 2000ns pulses and (b) 500ns pulses. “P#” represents the patient number and “T#” represents the tumor number as recorded on the patient’s records. Vertical dotted lines separate individual patients.

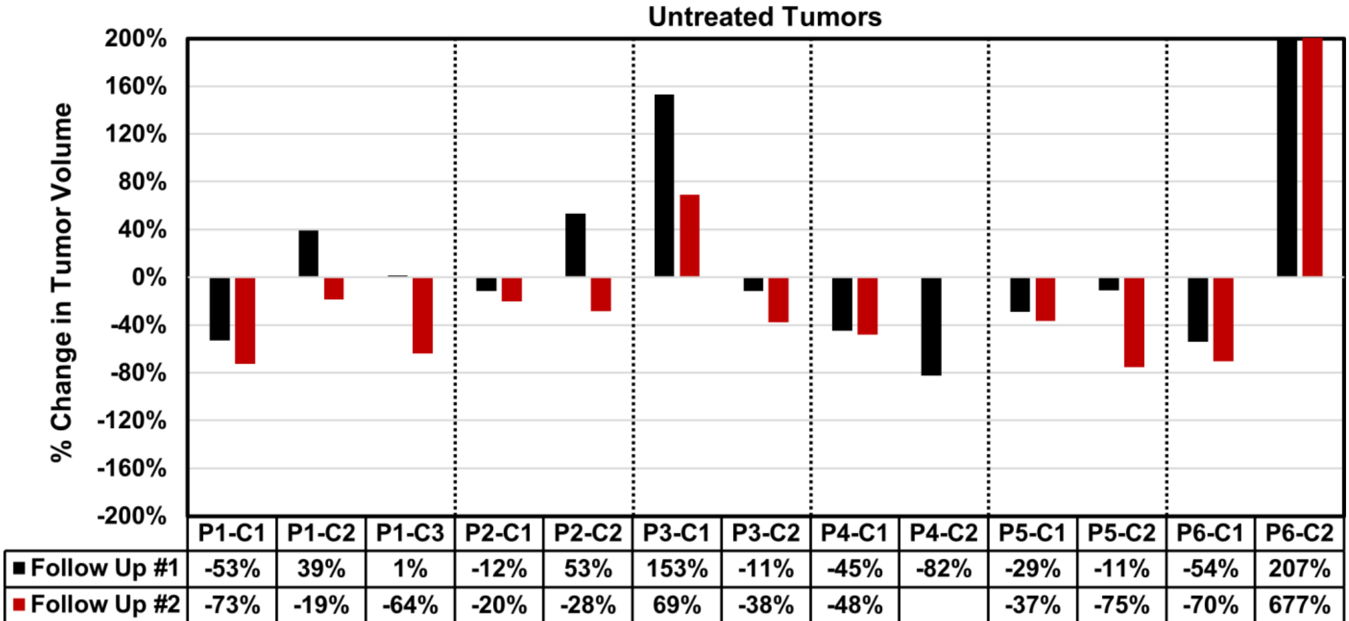

	P1-C1	P1-C2	P1-C3	P2-C1	P2-C2	P3-C1	P3-C2	P4-C1	P4-C2	P5-C1	P5-C2	P6-C1	P6-C2
■ Follow Up #1	-53%	39%	1%	-12%	53%	153%	-11%	-45%	-82%	-29%	-11%	-54%	207%
■ Follow Up #2	-73%	-19%	-64%	-20%	-28%	69%	-38%	-48%		-37%	-75%	-70%	677%

Supplemental Figure 8: Control Tumors Response. 13 untreated control tumors were tracked during the study. These tumors were in proximity to, but outside the treatment zone of other tumors treated in this study. "P#" represents the patient number and "C#" represents the untreated control tumor number as recorded on the patient's records. Vertical dotted lines separate individual patients.

Supplemental Video 1: **Response to INSPIRE Treatments.** INSPIRE Treatment of a tumor using the ring and pin applicator with a 2000V 2-5-2 waveform administered at a rate of 50 μs/s. The three sequential beeps at the beginning of the video indicate a three second countdown prior to pulses being delivered. Following this countdown, the animal's muscles tense slightly as the treatment is initiated. The subsequent bell-like sound is an indicator from the pulse generator that pulses are being delivered.

Tumor	Initial Tumor Volume [cm^3]	Final Tumor Volume [cm^3]	Voltage [V]	Dose [s]	Treatments
**Control Group**					
P1-T6	0.74	0.20	-	-	0
P1-T7	0.30	0.25	-	-	0
P1-T8	0.39	0.14	-	-	0
P2-T4	0.94	0.74	-	-	0
P2-T6	1.77	1.27	-	-	0
P3-T4	0.46	0.78	-	-	0
P3-T5	0.95	0.59	-	-	0
P4-T5	0.25	0.13	-	-	0
P4-T6	2.55	0.00	-	-	0
P5-T2	0.27	0.17	-	-	0
P5-T4	0.20	0.05	-	-	0
P6-T4	4.37	1.30	-	-	0
P6-T5	3.13	24.30	-	-	0
**500ns Treatments. Voltage: 1000V**					
P2-T3	0.43	0.16	1000	0.02	1
P2-T10	0.42	0.41	1000	0.02	1
P2-T11	0.36	0.35	1000	0.02	1
P3-T6	0.35	0.50	1000	0.02	2
P3-T7	0.21	0.15	1000	0.02	2
P3-T8	0.44	0.31	1000	0.02	2
**500ns Treatments. Voltage: 2000V**					
P4-T2	4.61	2.04	2000	0.02	1
P4-T3	4.22	0.18	2000	0.02	1
P5-T5	1.56	0.00	2000	0.02	1
**2000ns Treatments. Voltage: 1000-1500V**					
P2-T8	0.26	0.37	1000	0.02	1
P2-T9	0.44	0.37	1000	0.02	1
P3-T2	3.83	2.18	1000	0.02	2
P3-T3	0.85	0.29	1000	0.02	2
P6-T1	3.65	3.66	1000	0.02	2
P6-T2	2.49	2.96	1000	0.02	2
P6-T3	2.48	2.20	1000	0.02	2
P6-T6	12.99	4.20	1500	0.01	1
P6-T7	3.79	3.37	1500	0.01	1
**2000ns Treatments. Voltage: 2000V**					
P1-T1	2.37	1.76	2000	0.02	1
P1-T2	51.45	25.36	2000	0.02	2
P1-T4	1.66	0.00	2000	0.02	1
P1-T5	0.77	0.00	2000	0.02	1
P2-T1	7.63	0.00	2000	0.02	5
P2-T2	14.17	0.00	2000	0.02	5
P2-T5	1.61	0.00	2000	0.02	1
P3-T1	8.60	0.00	2000	0.02	5
P4-T1	2.16	0.00	2000	0.02	1
P4-T4	4.85	0.00	2000	0.02	1

Supplemental Table 1. Summary of tumors treated, treatment parameters, and treatment groups used for statistical analysis. The number of treatments indicates the number of overlapping treatments administered in a single visit as required to achieve total tumor coverage.
